# Diagnostic Challenges and Treatment Approach to Seronegative Autoimmune Encephalitis

**DOI:** 10.7759/cureus.56844

**Published:** 2024-03-24

**Authors:** Amman Bhasin, Alexis Haftka-George

**Affiliations:** 1 Internal Medicine, Thomas Jefferson University Hospital, Philadelphia, USA; 2 Internal Medicine, Henry Ford Health System, Detroit, USA

**Keywords:** immunology, neuroimmunology, encephalitis, autoimmune, paraneoplastic, seronegative autoimmune encephalitis

## Abstract

Seronegative autoimmune encephalitis (AE) is a rare, immune-mediated inflammatory syndrome that presents with a wide spectrum of neuropsychiatric symptoms, such as cognitive impairment, seizures, psychosis, focal neurological defects, and altered consciousness. This disease process presents with no identifiable autoimmune antibodies, which leads to uncertain diagnosis, delayed treatment, and prolonged hospital admissions. Early diagnosis and prompt treatment of AE should not be delayed, as early recognition and treatment leads to improved outcomes and disease reversibility for these patients. In this study, we present a case report of a 77-year-old male who presented with acutely altered mental status. This patient underwent an extensive workup and demonstrated no signs of clinical improvement throughout a prolonged hospital admission. The diagnostic challenges and treatment obstacles encountered during our care of this patient are described in this case report, along with recommendations for early diagnosis and prompt treatment for patients with suspected seronegative AE.

## Introduction

Autoimmune encephalitis (AE) diseases are non-infectious, immune-mediated inflammatory syndromes that result in rapid onset encephalopathy due to widespread brain inflammation [1-3]. AE is often a challenging clinical diagnosis due to its similarity in clinical presentation, laboratory results, and imaging findings of many AE subtypes and infectious causes of encephalitis [4]. Characteristic patient presentations of AE include a wide spectrum of acute to subacute neuropsychiatric symptoms including seizures, cognitive impairment, focal neurological defects, psychosis, and coma [2-5]. While previously described as a rare syndrome, recent epidemiological evidence shows that autoimmune encephalitis occurs at a similar frequency to infectious encephalitis with an estimated incidence of 13.7/100,000 cases [6].

The etiology of autoimmune encephalitis diseases occurs as a result of a host’s immune system targeting self-antigens expressed within the central nervous system (CNS) [1-3,7]. These AE diseases can be further divided into three broad subtypes: (1) autoimmune limbic encephalitis, (2) acute disseminated encephalomyelitis, and (3) antibody-negative probable autoimmune encephalitis (ANPRA). The etiology of these syndromes is a result of either paraneoplastic syndromes with antibodies against intracellular antigens, autoantibodies against extracellular receptors, ion channels, and proteins, or autoantibodies that have not been clearly established and have yet to be discovered [2-4,8].

Seronegative autoimmune encephalitis is a rare subtype of autoimmune encephalitis that occurs when there is no identifiable, pathologic autoantibody in either the cerebrospinal fluid (CSF) or serum of the patient. At present, the lack of identifiable autoantibodies in seronegative AE is hypothesized to be caused by unidentified autoantibodies that have not currently been discovered [8,9]. Ultimately, the lack of diagnostic testing to confirm seronegative AE results in diagnostic challenges and delayed treatment. This case report is presented to illustrate our experience with the diagnostic challenges associated with seronegative autoimmune encephalitis, along with proposed treatment and management options for these patients.

This article was previously presented as a meeting abstract and poster at the 2022 Society of General Internal Medicine (SGIM) Annual Meeting on April 9, 2022 in Orlando, Florida, USA.

## Case presentation

A 77-year-old male with a history of hypertension, type II diabetes mellitus, chronic kidney disease stage III, and hypothyroidism presented to the emergency department with acute altered mental status (AMS) after a reported episode of seizure-like activity. The patient was found lying on the ground adjacent to his farming tractor and was noted to have urinary incontinence, bilateral muscle jerking of both the upper and lower extremities, and was unresponsive for a total duration of 45 minutes witnessed by his family members. Following the episode, the patient began to experience cognitive dysfunction as his family members reported he was unable to recall his name, was unfamiliar with his home, and was unable to recognize any of his family members. After three hours, the patient continued to experience altered mentation and was brought to the hospital by EMS. Of note, prior to the initial episode of seizure-like activity, the patient was in his normal state of health and was described as independently able to carry out his activities of daily living.

Upon arrival to the emergency department, initial physical exam revealed word-finding difficulty and agitation, and the patient was oriented only to name with normal vital signs. Initial workup with computed tomography (CT) head imaging, CT angiography, chest X-ray, electroencephalogram (EEG) and urine toxicology was unremarkable. The neurology team was consulted to assess for a possible cerebrovascular accident (CVA) and an NIH Stroke Scale Score (NIHSS) of one was assigned, ruling out a possible CVA. Of note, the patient had mild leukocytosis, however, no electrolyte or other lab irregularities were noted. The patient was ultimately admitted for further workup and for continued AMS.

**Figure 1:**
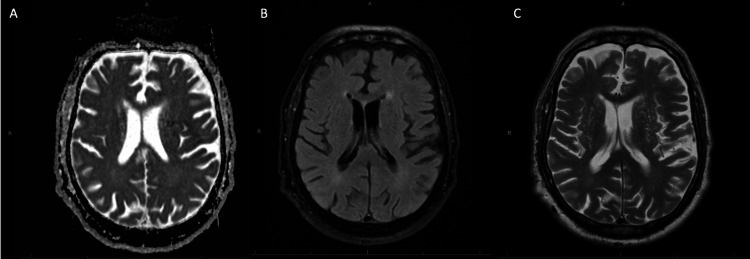
MRI Brain Imaging With and Without Contrast MRI Brain with and without contrast images demonstrating mild, chronic ischemic changes with no evidence of acute or subacute infarction. A: Diffusion Weighted Imaging (DWI), B: T1 weighted sequence, C: T2 fluid attenuated inversion recovery (FLAIR) sequence

On admission, further workup was significant for elevated free T4 levels and significantly low thyroid-stimulating hormone (TSH) levels despite the patient’s hypothyroid history. Serum studies were negative for thyroid peroxidase antibody (TPO), thyroglobulin antibody, antinuclear antibodies (ANA), double-stranded DNA (dsDNA), anti-Smith antigen, antineutrophil cytoplasmic antibodies (ANCA), C3/C4 complement levels, antiphospholipid antibody, anti-cardiolipin antibody, lupus anticoagulant, anti-SSA (Ro)/SSB (La), anti-RNP antibodies. Due to thyroid function abnormalities, the patient was promptly treated with methimazole and a euthyroid state was achieved. However, the patient continued to experience altered mental status, effectively ruling out a suspected thyroid-related encephalopathy diagnosis. Further neurological investigation revealed an unremarkable magnetic resonance imaging (MRI) of the brain (Figure 1), continuous EEG notable for diffuse background slowing with no seizure activity, and lumbar puncture (LP) revealed CSF studies notable for lymphocytic pleocytosis with infectious CSF panel negative for varicella zoster virus (VZV), herpes simplex virus (HSV), Epstein-Barr virus (EBV), West Nile virus, and cytomegalovirus (CMV). CSF autoimmune studies were positive for ANA, dsDNA antibodies, anti-histone antibodies, and elevated rheumatoid factor. Contrastingly, an extensive paraneoplastic and autoimmune panel returned negative for commonly detected autoantibodies, including anti-Hu, Ri, Yo, MA1, MA2, CV2, GAD, GlyR, NAE, GFAP, NMDA, AMPAR, MOG, ribosomal-P, S100B, aRNP, SSA, SSB, and thyroglobulin antibody all resulted negative. Negative results of these extensive paraneoplastic and autoimmune panels effectively rule out many possible neurological etiologies of encephalitis, including systemic lupus erythematosus (SLE) encephalitis and multiple known subtypes of autoimmune encephalitis. Relevant lab values are further described in Table 1.

**Table 1:** Relevant Laboratory Values T3/T4: thyroxine; TSH: thyroid stimulating hormone; WBC: white blood cell count

Pertinent Lab Values		
Lab Parameters (units)	Patient Lab Values	Reference Range
WBC (x 10^9^/L)	11.7	4.5 to 11.0
Free T4 (ng/dL)	3.34	0.8 to 1.8
TSH (mIU/L)	<0.01	0.5 to 5
First Lumbar Puncture		
Opening pressure (mmHg)	10	10-20
WBC (cells/mm^3^)	27 with 94% lymphocytes	<5
Protein (mg/dL)	345.4	15-40
Glucose (mg/100mL)	62	< 45
Second Lumbar Puncture		
Opening pressure (mmHg)	11	10-20
WBC (cells/mm^3^)	27 with 93% lymphocytes	<5
Protein (mg/dL)	185.4	15-40
Glucose (mg/100mL)	85	< 45

**Figure 2:**
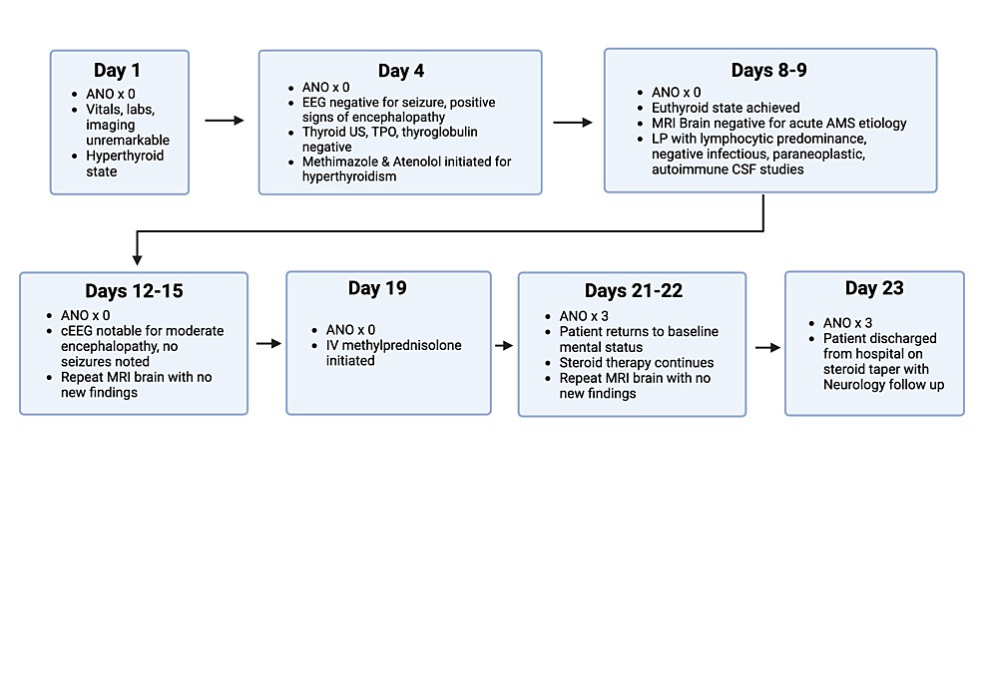
Timeline of Major Events and Clinical Course of Patient During Admission ANO: awake, alert, and oriented; ANO x 0: not oriented to person, place, or time; ANO x 3: alert and oriented to person, place, and time; CSF: cerebrospinal fluid; cEEG: continuous electroencephalogram; IV: intravenous; LP: lumbar puncture; MRI: magnetic resonance imaging; US: ultrasound

A diagnosis of seronegative autoimmune encephalitis was suspected and after 15 days of altered mentation and the patient was initiated on empiric steroid therapy with a five-day treatment course of 80 milligrams (mg) of oral prednisone. A repeat LP was conducted after the completed course of oral prednisone in order to determine whether the patient was responding to immunotherapy. Results of this second LP demonstrated signs of decreased inflammation, however a mild lymphocytic pleocytosis remained with a continued elevated protein level, which had decreased from the previous LP 10 days prior. Due to decreased inflammation, the decision was made to initiate the patient on a subsequent five-day treatment course of high-dose intravenous (IV) immunotherapy with 1 gram (g) IV methylprednisolone. After one day of IV steroid treatment, the patient demonstrated significant signs of improvement and rapidly returned to his baseline mental status after 20 days of hospital admission. The patient continued to demonstrate normal mentation and cognitive abilities while completing the course of steroids, and the patient was finally discharged after 23 days of admission. The timeline of events and clinical course of the patient are depicted in Figure 2, respectively.

## Discussion

Autoimmune encephalitis describes a group of disorders that produces widespread cerebral inflammation with a diverse set of clinical presentations that commonly include impaired cognitive ability, focal neurological defects, seizures, psychosis, and altered levels of consciousness. Seronegative autoimmune encephalitis, in particular, is a subtype of AE in which no identifiable autoantibody is detectable in either the serum or CSF of the patient, which can create diagnostic challenges [1-5,8].

The pathogenesis of AE has been categorized into three broad groups. The first group of AE is the paraneoplastic syndromes in which antibodies are created against intracellular tumor antigens such as anti-Hu, Yo, etc. This group of AE is strongly associated with cancer and involves T-cells attacking neuronal cells secondary to neoplasm targeting [3,4,8]. The second group of AE involves autoantibodies that target extracellular receptors, ion channels, or proteins of neuronal cells (i.e. MOG receptors). This group of AE is not typically associated with oncologic disease processes, unlike the paraneoplastic disease group. The third and final group of AE is comprised of autoantibodies that either have not clearly been established or have not been discovered at present [2-5,7,8].

Despite the increasing epidemiological evidence of AE diagnosis, there remain a number of diagnostic challenges and questions regarding the recommendations for diagnosis, treatment, and management of these patients. In one multicenter study, Baumgartner et al. found that an initial suspicion of an AE diagnosis was only suspected and confirmed in 32% of patients despite 80% of patients presenting with characteristic signs and symptoms that should have triggered suspicion for AE [10]. This case report presents a complex case of seronegative autoimmune encephalitis and illustrates the significant delays in diagnosis of AE, which ultimately leads to treatment delays and prolonged hospital admissions. Prompt recognition of characteristic clinical features of AE is imperative given the potential for reversibility of the condition with the correct treatment and management. In this case, once first-line treatment was initiated, the patient’s AE was rapidly reversed and significant improvement was achieved. Our recommendations for initial diagnosis, management, and treatment options for suspected AE patients are outlined below.

Autoimmune encephalitis is a diagnosis that should be considered by clinicians who encounter patients who present with an unexplained spectrum of neuropsychiatric symptoms that do not correspond to any vascular, infectious, toxic, or metabolic causes [4]. Current guidelines to definitively diagnose autoimmune encephalitis require confirmation of autoantibody presence or absence obtained from paraneoplastic, NMDA receptor, and other autoantibody panels that often cause significant delays in diagnosis and treatment [2,3]. Early recognition and initiation of treatment of seronegative encephalitis has been associated with improved patient outcomes, decreased mortality, and fewer cases of disease relapse [9,11,12]. It is our recommendation for clinicians suspicious of seronegative AE to utilize the criteria for ANPRA proposed by Graus et al. which state that ANPRA diagnosis can be confirmed when the following four criteria are all met: (1) a patient experiences rapid progression of memory deficits, AMS, or psychiatric symptoms within three months, (2) exclusion of well-defined syndromes of autoimmune encephalitis, (3) absence of defined autoantibodies in serum or CSF, and (4) reasonable exclusion of alternative causes [2]. These criteria rely largely on clinical history, physical exam findings, and the results of commonly available tests, allowing clinicians with a high suspicion of AE to prompt early recognition, management, and treatment independent of lengthy autoimmune antibody panels. However, it is important that serum and CSF autoantibody panels are still obtained, as their results ultimately inform the definitive diagnosis of autoimmune encephalitis, along with the specific subtype of AE diagnosis. This information is important for determination of possible underlying malignancy, long-term prognostic information, and guidance for treatment plans [2,3].

Once a high suspicion of a diagnosis of seronegative autoimmune encephalitis has been established, it is imperative that prompt immunotherapy treatment is initiated. Treatment recommendations have been outlined below, however, these recommendations have been derived from case series, case reports, and expert opinions, as no randomized control trials have been performed to corroborate optimal treatment guidelines. Recommended acute first-line therapy is with either (1) high-dose IV steroids (1g IV methylprednisolone) for a duration of three to seven days [2,3,12], (2) 1g IV methylprednisolone for three to five days in combination with intravenous immunoglobulin (IVIG) (0.4g/kg) for five days, (3) IVIG (2g/kg) for two to five days [3,12-14], or (4) plasmapheresis (PLEX) of five to 10 sessions every other day [3,12,15]. At present, there is no convincing evidence demonstrating the superiority of IVIG compared to plasmapheresis. If there is no clinical improvement after two weeks, second-line therapy should be initiated preferably with rituximab (375mg/m2 IV weekly) for four weeks or cyclophosphamide (750 mg/m2 IV monthly) for three to six months or until improvement is noted [3,4,12]. Rituximab is recommended over cyclophosphamide due to a more favorable safety profile [3,12].

## Conclusions

Seronegative autoimmune encephalitis is a disease subtype that has a suspected autoimmune etiology without an identifiable autoantibody in the serum or CSF, which can create diagnostic challenges, delayed treatment, and prolonged hospital admissions. Despite the recent discovery of new neuronal autoantibodies, many remain unidentified at present, thus a negative serology panel should not delay treatment in cases of suspected seronegative AE. High clinical suspicion of this disease should prompt clinicians to utilize clinical criteria to make a timely diagnosis of AE, followed by early initiation of immunotherapy (high dose steroids, IVIG, PLEX) to halt disease progression and reverse the inflammatory disease process. If no clinical improvements are noted, second-line treatment with rituximab or cyclophosphamide should be initiated.
